# Knowledge, attitude, and practices regarding climate change and its oral health effects among dental health care providers in India. A cross-sectional study

**DOI:** 10.21142/2523-2754-1403-2026-300

**Published:** 2026-07-10

**Authors:** M Vaishno Preethi, K Priya Deepa Lakshmi, M Muthu Sruthi, M Hemagayathri, Devi S Brinda, V Subhalakshmi

**Affiliations:** 1 Department of Public Health Dentistry, Vinayaka Mission’s Sankarachariyar Dental College, Vinayaka Mission’s Research Foundation (Deemed to be University). Salem, Tamil Nadu, India. vaishnopreethi@gmail.com, sruthimuthuvelu@gmail.com, hemagayathrislm@gmail.com Vinayaka Missions University Department of Public Health Dentistry Vinayaka Mission’s Sankarachariyar Dental College Vinayaka Mission’s Research Foundation (Deemed to be University) Salem, Tamil Nadu India vaishnopreethi@gmail.com sruthimuthuvelu@gmail.com hemagayathrislm@gmail.com; 2 Department of Environmental Science, Vinayaka Mission's College of Physiotherapy, Vinayaka Mission’s Research Foundation (Deemed to be University). Salem, Tamil Nadu, India. mokitha.10@gmail.com Vinayaka Missions University Department of Environmental Science Vinayaka Mission's College of Physiotherapy Vinayaka Mission’s Research Foundation (Deemed to be University) Salem, Tamil Nadu India mokitha.10@gmail.com; 3 Central Research Laboratory, Vinayaka Mission’s Sankarachariyar Dental College, Vinayaka Mission’s Research Foundation (Deemed to be University), Salem, Tamil Nadu, India. drvsubhalakshmi@vmsdc.edu.in Vinayaka Missions University Central Research Laboratory Vinayaka Mission’s Sankarachariyar Dental College Vinayaka Mission’s Research Foundation (Deemed to be University) Salem, Tamil Nadu India drvsubhalakshmi@vmsdc.edu.in

**Keywords:** climate change, dental students, oral health, cambio climático, estudiantes de odontología, salud bucodental

## Abstract

**Background:**

Climate change is increasingly recognized as a major public health threat. Despite its growing relevance, the implications for oral health and the preparedness of future dental professionals remain underexplored. This study assessed dental students' knowledge, attitude, and practices (KAP) regarding climate change and its effects on oral health.

**Materials and Methods:**

A cross-sectional study was conducted using a validated self-administered questionnaire comprising 29 closed-ended items across three KAP domains among dental students. Descriptive statistics summarized participant responses, while the Chi-square tests assessed associations between categorical variables, and independent t-tests and one-way ANOVA compared mean KAP scores across groups, with Tukey’s HSD for post hoc differences. A p-value < 0.05 was considered statistically significant.

**Results:**

More than half (55.8%) of students demonstrated good knowledge, with CRI students recording the highest mean knowledge score (11.78 ± 1.57). Knowledge scores differed significantly by year of study (p = 0.004). First-year students showed the highest attitude scores (8.52 ± 1.08; p < 0.001); however, 57.6% expressed unfavorable attitudes. Practices were the weakest domain, with only 45.6% exhibiting good climate-conscious behaviors (highest: first-year; 5.62 ± 1.61; lowest: third-year; 2.52 ± 2.07). Gender (p = 0.027) and year of study (p < 0.001) were significantly associated with practice scores. Significant positive correlations were found between KAP (p < 0.01), indicating mutually reinforcing domains.

**Conclusion:**

Strengthening curriculum integration and promoting climate-resilient behavior change among future dental professionals are essential to support environmentally responsible oral healthcare, ultimately ensuring quality care for the community.

## RESUMEN

**Antecedentes:**

El cambio climático se reconoce cada vez más como una importante amenaza para la salud pública. A pesar de su creciente relevancia, sus implicaciones para la salud bucodental y la preparación de los futuros profesionales de la odontología siguen siendo poco exploradas. Este estudio evaluó los conocimientos, actitudes y prácticas (CAP) de los estudiantes de odontología con respecto al cambio climático y sus efectos en la salud bucodental.

**Materiales y métodos:**

Se realizó un estudio transversal mediante un cuestionario autoadministrado validado, compuesto por 29 preguntas cerradas en tres dominios de CAP, entre estudiantes de odontología. Las estadísticas descriptivas resumieron las respuestas de los participantes, mientras que las pruebas de chi-cuadrado evaluaron las asociaciones entre variables categóricas, y las pruebas t independientes y el ANOVA unidireccional compararon las puntuaciones medias de CAP entre los grupos, utilizando la prueba HSD de Tukey para las diferencias post hoc. Un valor p < 0,05 se consideró estadísticamente significativo.

**Resultados:**

Más de la mitad (55,8 %) de los estudiantes demostraron un buen nivel de conocimientos, siendo los estudiantes de CRI los que registraron la puntuación media más alta (11,78 ± 1,57). Las puntuaciones de conocimiento difirieron significativamente según el año de estudio (p = 0,004). Los estudiantes de primer año mostraron las puntuaciones de actitud más altas (8,52 ± 1,08; p < 0,001); sin embargo, el 57,6 % expresó actitudes desfavorables. Las prácticas fueron el área más débil, con solo el 45,6 % mostrando buenos comportamientos de concienciación climática (mayor: primer año; 5,62 ± 1,61; menor: tercer año; 2,52 ± 2,07). El género (p = 0,027) y el año de estudio (p < 0,001) se asociaron significativamente con las puntuaciones de las prácticas. Se encontraron correlaciones positivas significativas entre los CAP (p < 0,01), lo que indica que los dominios se refuerzan mutuamente.

**Conclusión:**

Fortalecer la integración curricular y promover cambios de comportamiento resilientes al clima entre los futuros profesionales de la odontología es esencial para promover una atención bucodental responsable con el medio ambiente, garantizando así una atención de calidad para la comunidad.

## INTRODUCTION

Climate change refers to the long-term alteration of global or regional climate patterns, primarily driven by anthropogenic activities such as deforestation, industrialization, and the combustion of fossil fuels. This has a significant impact on ecosystems, human well-being, and environmental conditions, making it one of the greatest threats to public health. Variations in temperature, precipitation, and the frequency of extreme weather events compromise food and water security, contributing to increases in both communicable and noncommunicable diseases.

According to the Intergovernmental Panel on Climate Change (IPCC), over 3.6 billion people currently live in areas highly vulnerable to climate-related health impacts. The World Health Organization (WHO) estimates that climate change will result in approximately 250,000 additional deaths annually between 2030 and 2050 due to malnutrition, heat stress, diarrheal diseases, and vector-borne illnesses. ^2^ India is the 7th most affected country by climate change-induced extreme weather events, reporting 2,267 fatalities and substantial economic losses. ^3^ Several Indian studies have reported increasing health risks due to climate variability, a higher prevalence of vector-borne diseases in urban slums, and increasing mental health issues among vulnerable populations. ^4^^,^^5^

While the general health consequences of climate change are well-documented, its impact on oral health remains inadequately explored. Environmental changes can impact oral health through various mechanisms, including deteriorating water quality, altering dietary patterns, and disrupting access to dental care services during climate emergencies. Research has suggested that climate change may indirectly increase the risk of dental caries, periodontal disease, and oral infections. ^6^^,^^7^^,^^8^ In India, a study conducted in the southern region reported an increase in dental fluorosis linked to groundwater contamination resulting from changing rainfall patterns. ^9^ Another study from the Eastern Region of India revealed that over 55% of people in fluoride-endemic areas suffer from severe dental fluorosis, highlighting the climate-sensitive nature of groundwater quality. ^10^

Moreover, extreme weather events may hinder access to oral healthcare services, exacerbating the disease burden. Emerging research also emphasizes the ecological footprint of dental care. Duane et al. highlighted the significant carbon emissions associated with dental procedures and emphasized the need for sustainable practices in dental settings.^11^^)^ In the European region, a survey revealed that dental professionals were aware of dentistry’s contribution to climate change, indicating a substantial knowledge gap. ^12^ Another study conducted in Western India revealed that although students were somewhat aware of "green dentistry," its implementation was limited due to fiscal and institutional barriers. ^13^

Dental professionals, therefore, have a dual responsibility to minimize the environmental impact of clinical activities and to educate patients on the health risks associated with climate change. Despite growing concern, one scoping review has elucidated the knowledge and attitudes of staff regarding climate change and sustainability in dental practices. ^14^ In the context of occupational and environmental health, understanding the preparedness of future dental professionals is highly relevant, as dentistry is both affected by and contributes to environmental challenges. In contrast, no studies have been conducted on dental students-future dental professionals-regarding climate change and its implications for oral health. Hence, the research question is as follows:

What is the level of KAP regarding climate change and its effects on oral health among dental students in tertiary teaching hospitals?

Therefore, the present study aimed to assess KAP related to climate change and its oral health impacts among dental students. 

## MATERIALS AND METHODS

### Ethical considerations

The study received ethical approval from the Institutional Ethical Committee (VMSDC/IEC/Approval No. 433). Data confidentiality and voluntary participation were ensured.

### Study Design and Setting

An institution-based cross-sectional study was conducted over three months, from February 2025 to April 2025, at Vinayaka Mission’s Sankarachariyar Dental College (VMSDC), located in Salem, the first district to be formed in India and a rapidly urbanizing district in Tamil Nadu, which has experienced notable climate shifts in recent years, including rising temperatures, erratic monsoon patterns, and prolonged dry spells. These environmental changes affect water availability, groundwater quality, and overall public health. Such climate-related variations may also indirectly influence oral health by altering water fluoridation levels and dietary behaviours, during extreme weather events. 

### Study population

The study population comprised undergraduate (UG) dental students from all academic years: first year, second year, third year, fourth year, and compulsory rotatory internship (CRI) enrolled at the VMSDC. The students present during the data collection period were included, and those who declined to provide consent were excluded from the study. These criteria ensured that the participants represented the actively engaged student body and minimized nonresponse bias.

### Sampling Technique

A stratified random sampling technique was employed, wherein students were first divided into predefined strata based on their academic year, and participants were then randomly selected from each stratum in proportion to their population size to ensure adequate representation across all academic levels and minimize sampling bias. 

### Sample size determination

Sample size estimation was performed using the finite population correction formula, as the study population comprised 433 total dental students. In the absence of prior empirical estimates for the primary outcome variables (KAP scores), the anticipated population proportion was conservatively set at 0.5 to maximize variance and yield the largest required sample size. The allowable absolute precision was fixed at 5%, and a two-sided confidence level of 95% was applied, corresponding to a standard normal deviate of 1.96. Given the use of stratified random sampling and the assumption of minimal variance inflation across strata, the design effect was set as 1. Incorporation of these parameters into the finite population formula resulted in a minimum estimated sample size of 204 participants.

### Data collection tool and procedure

The first section of the questionnaire was designed to record the general information (age, gender, and year of study) of each participant. The second section of the questionnaire focused on assessing students' KAP regarding climate change and its influence on oral health. This section consisted of 29 closed-ended questions grouped into three domains:

1. Knowledge Domain (13 items): These items evaluate students’ understanding of climate change, its anthropogenic causes, links to environmental pollution, and its direct or indirect impacts on oral health. The key areas included awareness of the Environmental Protection Act, effects of water contamination, exposure to carbon emissions, and associations with dental conditions such as dental caries, lip cancer, and temporomandibular disorders (TMDs).
2. Attitude domain (9 items): These questions gauged the participants’ perceptions of climate change as a global and oral health concern, their concern for future generations, their belief in the role of dentists in climate education, and their acceptance of eco-friendly practices and digital dentistry as part of climate action.
3. Practice Domain (7 items): This section assessed proactive behaviors, including learning about climate-related oral health issues, participating in awareness campaigns, providing advice on preventive strategies, and engaging in environmental pledges.

All the KAP items were scored on a dichotomous scale: "Yes" = 1, "No" = 0. Higher cumulative scores indicate good knowledge, positive attitudes, and good environmentally conscious practices.

### Questionnaire validation and reliability

The initial draft of the questionnaire was developed following an in-depth review of the relevant scientific literature on climate change, environmental health, and oral health. To establish content and face validity, the questionnaire was independently reviewed by a panel of three subject experts, including a public health dentist, an environmental health researcher, and a faculty member in dental education. Their input was used to refine the clarity, relevance, and logical flow of the questions.

To assess the internal consistency of the questionnaire, a reliability analysis was conducted via Cronbach’s alpha coefficient. The analysis yielded the following results: overall Cronbach’s alpha = 0.82, indicating good internal consistency [knowledge domain (13 items) = 0.78, attitude domain (9 items) = 0.81, practice domain (7 items) = 0.76].

### Statistical analysis

The IBM Statistical Package for Social Sciences version 26 (SPSS Inc., Chicago, IL, USA) software) was used to perform descriptive and inferential statistics. Descriptive statistics were used to summarize the general information of the participants and their responses to the KAP items. To assess the associations between demographic variables and KAP scores, the chi-square test was applied to examine the relationships between categorical variables. Independent t-tests were used to compare the mean KAP scores between the two groups. One-way ANOVA was used to compare the mean scores across multiple academic years. Post hoc analysis, using Tukey’s HSD test, was then performed to identify specific groupwise differences. A p-value < 0.05 was considered statistically significant.

## RESULTS

A total of 433 dental students were invited to participate; of these, 342 completed the questionnaire, yielding a response rate of 79.5%. The distribution of respondents closely reflected the planned strata, with first-year students (n = 77, 22.5%) and CRIs (n = 76, 22.2%) forming the largest groups, followed by third-year (n = 67, 19.6%), fourth-year (n = 63, 18.4%), and second-year students (n = 59, 17.3%). The sample was predominantly female (n = 241, 70.5%), while males accounted for 29.5% (n = 101). The mean age of participants was 20.9 ± 1.82 years.

The overall mean knowledge score among CRI participants was 11.78 ± 1.571, indicating a relatively good understanding of climate change and its implications for oral health, which reflects more comprehensive knowledge gained during training. When asked whether *“climate change is due to natural factors and is a recent phenomenon”* (Q4), the responses varied significantly (p < 0.001) by year (Table 1). This reflects an improved understanding of the anthropogenic causes of climate change among senior students. However, when *“Can climate change increase the risk of dental caries in children?”* (Q8), many fourth-year (0.57 ± 0.499) and CRI (0.74 ± 0.443) students responded incorrectly, indicating notable gaps in understanding the indirect pathways through which climate change may influence oral health, such as altered dietary patterns, increased consumption of processed and sugary foods during climate-related stress, disruptions in water quality and fluoride availability, and reduced access to preventive dental care. The question (Q2) *“Do you accept that environmental pollution affects climate change?”* received the highest score across all participants (1.000 ± 0.000), reflecting universal agreement (Table 1).

**Table 1:** Mean scores for KAP regarding climate change and its oral health effects stratified by study participants' year of study

KAP items	Year of Study			
	First Year (Mean ± SD)	Second Year (Mean ± SD)	Third Year (Mean ± SD)	Fourth Year (Mean ± SD)	CRI (Mean ± SD)	p value
1. Are you aware that climate change and Environmental pollution are interrelated?	0.92 ± 0.270	0.92 ± 0.281	0.97 ± 0.171	0.98 ± 0.126	0.97 ± 0.161	0.195
2. Do you accept that environmental pollution alters the climate?	0.92 ± 0.270	1.00 ± 0.000	0.99 ± 0.122	1.00 ± 0.000	0.97 ± 0.160	0.020*
3. Are humans responsible for climate change?	0.97 ± 0.160	0.97 ± 0.183	0.91 ± 0.288	1.00 ± 0.000	0.97 ± 0.161	0.074
4. Is climate change due to natural factors a recent phenomenon?	0.60 ± 0.494	0.68 ± 0.471	0.54 ± 0.502	0.87 ± 0.336	0.92 ± 0.271	< 0.001*
5. Are you aware of the Environmental Protection Act 1986?	0.90 ± 0.307	0.93 ± 0.254	0.84 ± 0.373	0.86 ± 0.353	0.89 ± 0.309	0.487
6. Have you observed or learned that water contamination can affect Oral health?	0.95 ± 0.223	0.98 ± 0.130	0.97 ± 0.171	0.95 ± 0.215	0.92 ± 0.271	0.494
7. Prolonged exposure to high levels of carbon emissions can heighten the risk of oral diseases.	0.94 ± 0.284	0.83 ± 0.378	0.76 ± 0.430	0.84 ± 0.368	0.86 ± 0.354	0.071
8. Can climate change increase the risk of dental caries in children?	0.97 ± 0.160	0.71 ± 0.457	0.69 ± 0.467	0.57 ± 0.499	0.74 ± 0.443	< 0.001*
9. Does cold weather have a significant impact on sinus issues?	0.96 ± 0.195	0.98 ± 0.130	0.85 ± 0.359	0.94 ± 0.246	1.00 ± 0.000	< 0.001*
10. Prolonged exposure to the sun’s ultraviolet radiation can increase the risk of developing lip cancer.	0.96 ± 0.195	0.95 ± 0.222	0.88 ± 0.327	0.98 ± 0.126	0.99 ± 0.115	0.023*
11. Will low climate temperatures be associated with an increase in dental pain or Temporomandibular Disorders (TMD)?	0.94 ± 0.248	0.80 ± 0.406	0.69 ± 0.467	0.75 ± 0.439	0.88 ± 0.325	< 0.001*
12. Can Climate change and antimicrobial resistance (AMR) be related?	0.74 ± 0.441	0.78 ± 0.418	0.76 ± 0.430	0.83 ± 0.383	0.82 ± 0.390	0.708
13. Do the causes of climate change directly or indirectly impact the field of dentistry?	0.95 ± 0.338	0.86 ± 0.345	0.90 ± 0.308	0.92 ± 0.272	0.84 ± 0.367	0.683
Total Knowledge Score	11.64 ± 1.413	11.39 ± 1.691	10.73 ± 1.638	11.49 ± 1.162	11.78 ± 1.571	< 0.001*
14. Do you think that climate change is a global emergency?	0.95 ± 0.223	0.97 ± 0.183	0.94 ± 0.239	0.94 ± 0.246	1.00 ± 0.000	0.292
15. Do you think climate change will affect future generations' oral health?	0.95 ± 0.223	0.88 ± 0.326	0.85 ± 0.359	0.89 ± 0.317	0.89 ± 0.311	0.433
16. Are you concerned about climate change and its potential influence on Oral Health?	0.97 ± 0.160	0.88 ± 0.326	0.90 ± 0.308	0.90 ± 0.296	0.97 ± 0.161	0.075
17. Do you believe that climate change is a factor in dentinal hypersensitivity?	0.94 ± 0.248	0.71 ± 0.457	0.72 ± 0.454	0.78 ± 0.419	0.93 ± 0.250	< 0.001*
18. Do you think that quitting smoking is a climate change intervention?	0.96 ± 0.195	0.61 ± 0.492	0.57 ± 0.499	0.65 ± 0.481	0.87 ± 0.340	< 0.001*
19. Do you accept that sustainable environmental (eco-friendly) practices can reduce long-term oral health issues?	0.92 ± 0.270	0.88 ± 0.326	0.88 ± 0.327	0.90 ± 0.296	0.93 ± 0.250	0.761
20. Does the dentist have a role in educating patients about the effects and preventive strategies of oral health towards climate change?	0.97 ± 0.160	0.93 ± 0.254	0.88 ± 0.327	0.95 ± 0.215	0.89 ± 0.309	0.170
21. Do you agree that it is important to understand this climate change issue, which will help in patient care?	0.95 ± 0.223	0.88 ± 0.326	0.91 ± 0.288	0.97 ± 0.177	0.89 ± 0.309	0.310
22. Do you agree that digital dental technology reverses climate change?	0.91 ± 0.289	0.56 ± 0.501	0.54 ± 0.502	0.43 ± 0.499	0.57 ± 0.499	< 0.001*
Total Attitude score	8.52 ± 1.083	7.31 ± 1.704	7.81 ± 2.044	7.41 ± 1.375	7.95 ± 1.285	< 0.001*
23. Have you learned how environmental climate change might influence the prevalence of oral diseases?	0.88 ± 0.323	0.44 ± 0.501	0.46 ± 0.502	0.65 ± 0.481	0.61 ± 0.492	< 0.001*
24. Have you ever discussed climate change and its oral health effects with your colleagues/family members/friends, or any others?	0.87 ± 0.338	0.49 ± 0.504	0.37 ± 0.487	0.57 ± 0.499	0.61 ± 0.492	< 0.001*
25. Did you ever encounter an oral health issue caused by climate change?	0.26 ± 0.441	0.08 ± 0.281	0.09 ± 0.288	0.19 ± 0.396	0.34 ± 0.478	< 0.001*
26. Have you ever noticed or been informed by your family members/relatives/friends or any others of an oral health issue due to climate change?	0.91 ± 0.289	0.42 ± 0.498	0.21 ± 0.410	0.48 ± 0.504	0.45 ± 0.501	< 0.001*
27. Have you ever been involved/organized/participated in environmental-related awareness campaigns or competitions?	0.92 ± 0.270	0.56 ± 0.501	0.42 ± 0.497	0.54 ± 0.502	0.51 ± 0.503	< 0.001*
28. Have you ever advised preventive strategies for an oral health issue caused by climate change to your family members/relatives/friends, or any others?	0.92 ± 0.270	0.42 ± 0.498	0.39 ± 0.491	0.41 ± 0.496	0.49 ± 0.503	< 0.001*
29. Have you pledged/signed to protect an environmental issue?	0.84 ± 0.365	0.49 ± 0.504	0.58 ± 0.497	0.63 ± 0.485	0.49 ± 0.503	< 0.001*
Total Practice score	5.62 ± 1.606	2.92 ± 2.307	2.52 ± 2.070	3.48 ± 2.292	3.49 ± 2.646	< 0.001*

CRI-Compulsory Rotatory Internship, *Significance level of p < 0.05, SD - Standard Deviation

In the attitude section, the items that demonstrated statistically significant differences (*p* = 0.001) included *the following: Q17- “Do you believe that climate change contributes to dentinal hypersensitivity?”, Q18- “Do you consider smoking cessation a climate change intervention?”, and Q22- “Can digital dental technologies help reverse climate change?”* These findings highlight that younger students held more favorable views regarding the impact of climate change on their oral health and were more accepting of nontraditional interventions. The attitude scores differed significantly across academic years (p < 0.001), with first-year students reporting the highest average score (8.52 ± 1.083) (Table 1).

Practice behaviors were less favorable across the group. First-year students reported the highest practice scores (5.62 ± 1.606), whereas third-year students had the lowest (2.52 ± 2.070). The responses to questions such as Q27-*“Have you ever participated in environmental-related awareness campaigns or competitions?”*, Q28-*“Have you advised preventive strategies for oral health issues caused by climate change to your family members/friends/any others?”*, and *Q29-“Have you pledged to protect an environmental issue?”* presented significant yearwise variation (p < 0.001), indicating that engagement with environmental practices was highest among early-year students (Table 1). When we asked about oral health problems, very few (N = 47, 13.7%) encountered issues caused by climate change.

In total, 55.8% of the students demonstrated good knowledge, whereas 44.2% had poor knowledge. The knowledge scores were significantly different across academic years (p = 0.004), with first-year and CRI students scoring better than second, third-, and fourth-year students. The attitude domain revealed that 57.6% of the participants had a negative attitude, whereas 42.4% had a positive attitude. Only 45.6% of the students reported good practices, whereas 54.4% reported poor practices. There were statistically significant differences based on gender (p = 0.027) and year of study (p < 0.001). Females and first-year students were more engaged in climate-friendly actions.

In our study, we went one step ahead and compared the KAP scores among all the study participants and reported that the mean knowledge score was 11.42 ± 1.53 (95% CI: 11.26-11.58), indicating that the participants had a relatively good level of knowledge with low variability among the responses. The mean attitude score was 7.72 ± 1.59 (95% CI: 7.55-7.89), reflecting a generally positive attitude. The narrow confidence interval shows consistency in the responses, and the highly significant p-value (<0.001) indicates that the result is robust. Compared with the KAP scores (3.68 ± 2.46 [95% CI: 3.42-3.94], p < 0.001), which were lower and showed greater variability, this suggests that although the participants were aware of and held positive attitudes, these were not fully translated into consistent practices (Table 2).

**Table 2:** Descriptive statistics and significance of KAP scores among study participants

Variable	Mean ± SD	95% Confidence Interval		P value
		Lower Bound	Upper bound	
Knowledge	11.42 ± 1.527	11.26	11.58	< 0.001*
Attitude	7.72 ± 1.591	7.55	7.89	< 0.001*
Practice	3.68 ± 2.457	3.42	3.94	< 0.001*
KAP	25.77 ± 4.754	25.27	26.28	< 0.001*

*Significance level of P<0.05, SD - Standard Deviation

To identify specific group differences, the post hoc pairwise comparison revealed that first-year students had the lowest KAP scores, which were significantly (p < 0.001) lower than those of all other years. KAP scores improved with progression, peaking around the third year and the CRI. The most consistent gap was between first-years and all higher levels, underscoring the role of academic exposure and clinical training (Table 3).

**Table 3:** Post-hoc pairwise comparison of KAP scores by year of study

(I) Year of Study	(J) Year of Study	Mean Difference (I-J)	Sig.	95% Confidence Interval	
				Lower Bound	Upper Bound
First Year	Second Year	4.60*	< 0.001*	2.56	6.64
	Third Year	5.97*	< 0.001*	4.00	7.95
	Fourth Year	3.92*	< 0.001*	1.91	5.92
	CRI	2.85*	< 0.001*	.94	4.76
Second Year	First Year	-4.60*	< 0.001*	-6.64	-2.56
	Third Year	1.37	0.384	-.74	3.48
	Fourth Year	-.68	0.906	-2.82	1.46
	CRI	-1.75	0.136	-3.80	.30
Third Year	First Year	-5.97*	< 0.001*	-7.95	-4.00
	Second Year	-1.37	0.384	-3.48	.74
	Fourth Year	-2.06	0.053	-4.13	.02
	CRI	-3.12*	< 0.001*	-5.10	-1.14
Fourth Year	First Year	-3.92*	< 0.001*	-5.92	-1.91
	Second Year	.68	0.906	-1.46	2.82
	Third Year	2.06	0.053	-.02	4.13
	Fourth Year	-1.06	0.597	-3.08	.95
CRI	First Year	-2.85*	< 0.001*	-4.76	-.94
	Second Year	1.75	0.136	-.30	3.80
	Third Year	3.12*	< 0.001*	1.14	5.10
	Fourth Year	1.06	0.597	-.95	3.08

The error term is mean square (error) = 18.555.

*The mean difference is significant at the .05 level.

When comparing the level of KAP between the years of study, there was a statistically significant variation in knowledge (p = 0.004), attitude (p < 0.001), and practice (p < 0.001) scores across different years of study. First-year and CRI students demonstrated comparatively better knowledge and attitudes, while practice levels were lower in the intermediate academic years, highlighting a notable knowledge-practice gap (Table 4).

**Table 4:** Comparison of the level of KAP between the years of study

Year of Study	Knowledge		Attitude			Practice	
	Poor	Good	Poor	Good	Poor	Good
	N (%)	N (%)	N (%)	N (%)	N (%)	N (%)
First year	31(20.5)	46(24.1)	19(9.6)	58(40.0)	10(5.4)	67(42.9)
Second year	24(15.9)	35(18.3)	42(21.3)	17(11.7)	43(23.1)	16(10.3)
Third year	43(28.5)	24(12.6)	45(22.8)	22(15.2)	51(27.4)	16(10.3)
Final year	28(18.5)	35(18.3)	48(24.4)	15(10.3)	38(20.4)	25(16.0)
CRI	25(16.6)	51(26.7)	43(21.8)	33(22.8)	44(23.7)	32(20.5)
P value	0.004*		< 0.001*		< 0.001*	

*Significance level of p < 0.05

## DISCUSSION

This institution-based cross-sectional study assessed the KAP of dental students concerning climate change and its impact on oral health. The findings indicate a relatively high level of awareness among students but reveal gaps in attitudes and practical engagement, particularly during the mid-academic years.

The overall mean knowledge score indicates that the CRI participants had a sound general understanding of the concept of climate change. This finding is consistent with those of Leal Filho et al. ^15^, who reported similar awareness levels among university students across multiple countries. In India, Ramya Kundayi Ravi et al. ^16^ reported comparable results among nursing students.

Interestingly, in our study, only one-fourth of the CRI and first-year students recorded good knowledge scores. This may be attributed to recent exposure to environmental science topics in the first year and enthusiasm for broader public health themes. However, knowledge about specific oral health effects of climate change-such as its influence on dental caries, mucosal conditions, or temporomandibular disorders-is relatively poor. This finding is consistent with recent evidence, highlighted by Bhadauria et al., ^17^ who reported that the relationship between climate change and oral health outcomes remains unrecognized and underresearched.

The highest mean score was observed for the question *“Do you accept that environmental pollution affects climate change?”* (1.000 ± 0.000), which aligns with the general public’s understanding, given the influence of media and education campaigns. However, lower scores on items related to oral-specific outcomes demonstrate fragmented climate-health literacy in dental education.

Despite relatively strong knowledge, attitudes were poor in more than half of the participants, which does not concur with the findings of the study by Haque et al. ^18^ on the population of Saudi Arabia. First-year students had more positive attitudes, whereas second- to fourth-year students displayed more negative attitudes. This attitudinal stagnancy may reflect curriculum fatigue or a lack of reinforcement as students progress, a trend also observed by Gupta et al. ^19^

Low scores for attitude items such as *“quitting smoking as a climate action”* and *“digital dentistry reduces environmental impact”* reflect limited interdisciplinary integration of climate change in the dental curriculum, revealing that dental education often lacks environmental modules, leaving students unable to link clinical behaviors with sustainability goals. Internationally, Ccami-Bernal et al. ^20^ and Khabour et al. ^21^ also emphasized that without structured curricula, health students perceive climate change as abstract and unrelated to their clinical responsibilities.

The weakest domain was practice, where only 45.6% of the students demonstrated climate-responsible actions, such as attending environmental campaigns, advising others, or advocating sustainable habits. This gap is consistent with the findings of an Indian study carried out in every zonal region by Sambath et al. ^(²²)^, who demonstrated that the healthcare workforce possessed good theoretical knowledge but that very few engaged in sustainable activities. However, they also noted a lack of inclusion of the dental team, highlighting the gap in addressing oral health within the broader healthcare response to climate change. Reddy et al. ^23^ reported a similar discrepancy in eco-dental practices in India.

Interestingly, our study revealed that first-year students had better environmental practices than senior students, echoing the findings of Sulistyawati et al. ^24^, who reported that younger generations are more responsive to new social and environmental messages. The results suggest a need for vertical integration of climate change themes throughout all years of dental education. This "knowledge‒practice gap" supports F.A. Perotti et al. ^25^ and Y Kurt et al. ^26^, who concluded that knowledge must be paired with motivation, role modeling, and active learning strategies to translate into behavior change.

Our findings revealed significant positive correlations between KAP scores, indicating that improvements in knowledge are likely to enhance students’ attitudes and practices. The health belief model and social cognitive theory support this linkage by emphasizing the role of perceived relevance and environmental cues in triggering behavioral changes. ^27^

### Strengths and Limitations

A key strength of this study is its broad inclusion of dental students across all academic levels and its focus on climate change-a topic that is largely underrepresented in oral health research. The use of a validated questionnaire and a strong response rate (79.5%) further strengthens its reliability. However, limitations include its single-institution scope, limited generalizability, and the use of self-reported data, which may introduce response bias.

## CONCLUSIONS

Dental students possessed relatively good knowledge and moderately positive attitudes toward climate change and oral health, these attitudes did not consistently translate into sustainable practices. Strengthening dental curricula, enhancing institutional infrastructure that supports environmentally responsible clinical activities, and integrating experiential, practice-oriented training is essential to bridge the knowledge-practice gap and prepare future dentists for climate-resilient oral healthcare, ultimately ensuring quality care for the community.

## Sources of financing:

Nill
